# A genetic mosaic mouse model illuminates the pre-malignant progression of basal-like breast cancer

**DOI:** 10.1242/dmm.050219

**Published:** 2023-11-13

**Authors:** Jianhao Zeng, Shambhavi Singh, Xian Zhou, Ying Jiang, Eli Casarez, Kristen A. Atkins, Kevin A. Janes, Hui Zong

**Affiliations:** ^1^ Department of Microbiology, Immunology, and Cancer Biology, University of Virginia Health System, Charlottesville, VA 22908, USA; ^2^ Department of Biomedical Engineering, University of Virginia, Charlottesville, VA 22908, USA; ^3^ Department of Pathology, University of Virginia Health System, Charlottesville, VA 22908, USA; ^4^ University of Virginia Comprehensive Cancer Center, University of Virginia Health System, Charlottesville, VA 22908, USA

**Keywords:** Mouse genetic mosaic model, Basal-like breast cancer, *BRCA1*, Pre-malignancy, Spatiotemporal analysis of tumor initiation

## Abstract

Basal-like breast cancer (BLBC) is highly aggressive, and often characterized by *BRCA1* and *p53* deficiency. Although conventional mouse models enabled the investigation of BLBC at malignant stages, its initiation and pre-malignant progression remain understudied. Here, we leveraged a mouse genetic system known as mosaic analysis with double markers (MADM) to study BLBC initiation by generating rare GFP^+^ *Brca1*, *p53*-deficient mammary cells alongside RFP^+^ wild-type sibling cells. After confirming the close resemblance of mammary tumors arising in this model to human BLBC at both transcriptomic and genomic levels, we focused our studies on the pre-malignant progression of BLBC. Initiated GFP^+^ mutant cells showed a stepwise pre-malignant progression trajectory from focal expansion to hyper-alveolarization and then to micro-invasion. Furthermore, despite morphological similarities to alveoli, hyper-alveolarized structures actually originate from ductal cells based on twin-spot analysis of GFP-RFP sibling cells. Finally, luminal-to-basal transition occurred exclusively in cells that have progressed to micro-invasive lesions. Our MADM model provides excellent spatiotemporal resolution to illuminate the pre-malignant progression of BLBC, and should enable future studies on early detection and prevention for this cancer.

## Teaser

**Editor's choice:** A mouse model that reveals the process of basal-like breast cancer initiated from sporadic *Brca1, p53*-deficient cells enables spatially resolved analysis of the pre-malignant progression of mutant cells.

## INTRODUCTION

Breast cancer is the most frequently diagnosed cancer type and the second leading cause of cancer death in those assigned female at birth (Siegel et al., 2022). Human breast cancer is a heterogeneous disease classified into six molecular subtypes with distinct prognosis: luminal A, luminal B, HER2-enriched, normal-like, claudin-low and basal-like (Perou et al., 2000; Prat et al., 2010; Tobin et al., 2015). Basal-like breast cancer (BLBC) accounts for 15-20% of breast cancer cases and is the most aggressive subtype, with earlier onset, increased chance of metastasis and absence of hormonal-therapy targets (Fulford et al., 2007; Millikan et al., 2008; Tobin et al., 2015; Turner et al., 2004). BLBCs show a high prevalence of *p53* (also known as *TP53* in human) mutations (∼80%) and deficiency in homology-directed DNA repair (∼50%); the latter is often caused by germline mutations in *BRCA1/2*, somatic epigenetic inactivation of *BRCA1/2*, or the loss of other essential genes for homology-directed DNA repair pathway (collectively termed as ‘BRCAness’) (Lord and Ashworth, 2016; McCabe et al., 2006; Network, 2012; Tian et al., 2019). Early detection and prevention of BLBC can fundamentally improve patient care, particularly for individuals with germline *BRCA1* mutations who are at a high risk of BLBC. Therefore, it is imperative to gain a deep understanding of how BLBC initiates and progresses during the pre-malignant stages.

Genetically engineered mouse models (GEMMs) present an invaluable pre-clinical platform for studying human cancers. Conditional knockout of *Brca1* and *p53* (also known as *Trp53* in mouse) in mouse mammary epithelial cells led to mammary tumors resembling human BLBC (Hollern et al., 2019; Liu et al., 2007; Molyneux et al., 2010; Xu et al., 1999). These models are useful for studying BLBC at the malignant stage, but are quite limited in examining cancer initiation and pre-malignant progression. First, conditional knockout models generate numerous rather than rare mutant cells at the cancer initiation stage; thus, they do not accurately mimic human cancer initiation from sporadic mutant cells (Liu et al., 2011; Muzumdar et al., 2007), which may impact pre-malignant development. Second, even if rare mutant cells can be generated, unequivocally pinpointing subtle aberrant behaviors of *BRCA1* mutant cells at the pre-malignant stage remains challenging.

**Fig. 1.**
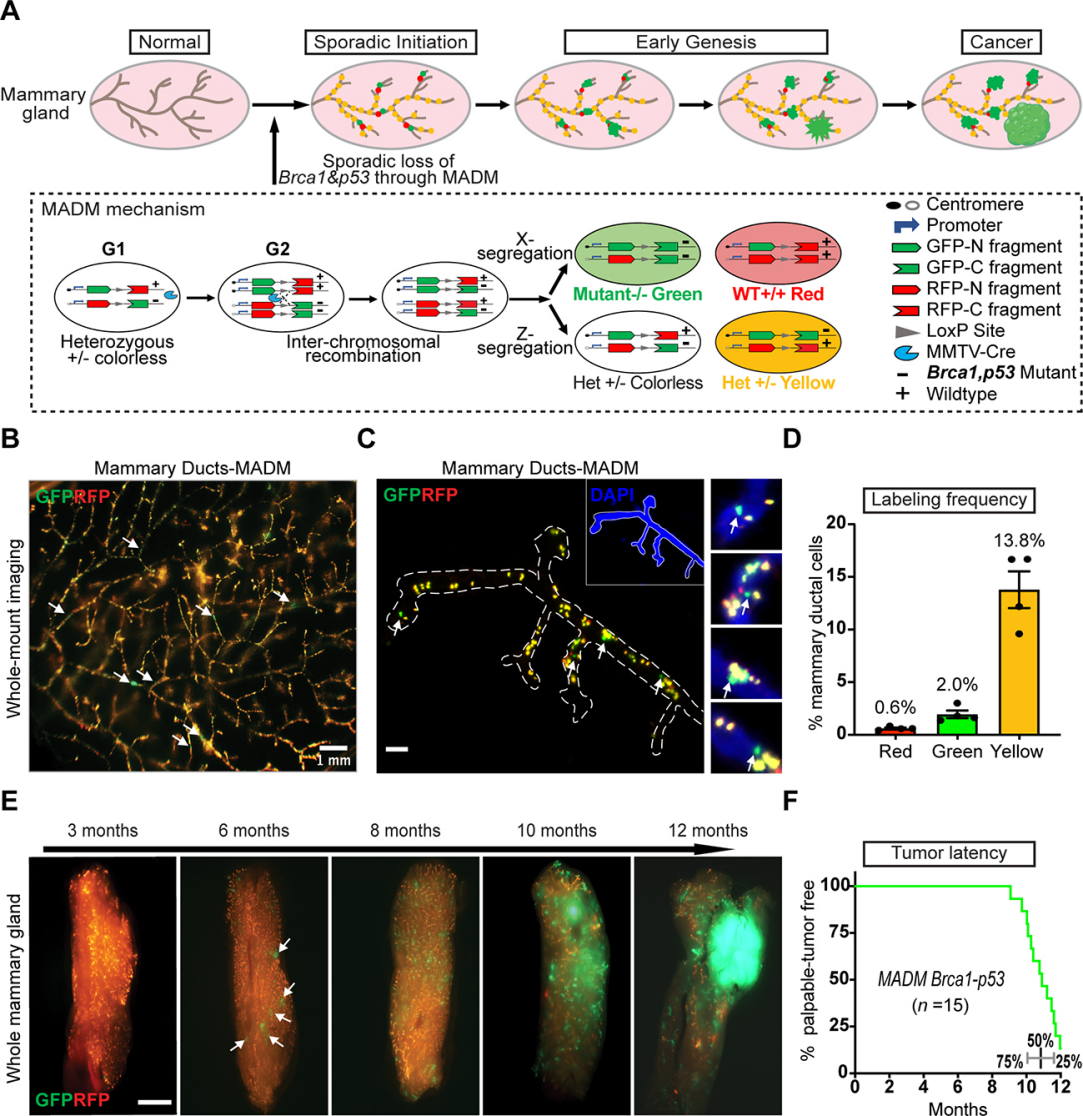
**A mosaic analysis with double markers (MADM) model that tracks the process of *Brca1, p53*-dependent mammary tumorigenesis from sporadic cancer-initiating cells to frank tumors.** (A) Modeling breast cancer development from sporadic mutant cells to tumors with MADM. From a colorless heterozygous mouse, through inter-chromosomal recombination in mitotic cells at the G2 phase, MADM generates one GFP^+^ mutant cell and one RFP^+^ wild-type cell after X segregation (two recombinant sister chromatids segregate into different daughter cells); alternatively, Z segregation generates one colorless and one dual-colored (yellow) cell that are both heterozygous (two recombinant sister chromatids segregate into the same daughter cells). The MADM schematic is reproduced with permission from Zong et al. (2005). This image is not published under the terms of the CC-BY license of this article. For permission to reuse, please see Zong et al. (2005). (B) The MADM model induces sparse and scattered GFP^+^ mutant cells (arrows) in mouse mammary glands. The image is representative of four MADM-mutant mice collected at 3 months of age for whole-mount fluorescence imaging. Scale bar: 1 mm. (C) High-resolution imaging of the GFP^+^ mutant cells (arrows) in mammary glands from 3-month-old MADM-mutant mice. The dashed line outlines the area of mammary ducts. The image is representative of mammary tissue sections acquired from four mice at 3 months of age and imaged with wide-field fluorescence microscopy. Scale bar: 100 μm. (D) The proportion of MADM-labeled cells among all mammary epithelial cells in MADM-mutant mice at 3 months of age. Sections of mammary glands were imaged with wide-field microscopy. The number of MADM-labeled and total mammary ductal cells was counted by GFP/RFP fluorescence and DAPI, respectively. Data are shown as the mean percentage±s.d. from *n*=4 mice. (E) Fluorescence imaging of whole mammary glands from a cohort of MADM-mutant mice at different ages, showing the progressive expansion of GFP^+^ mutant cells toward tumor formation. Arrows indicate GFP^+^ focal expansions. Images were collected on a fluorescence stereomicroscope and are representative of ten mice for each age. Scale bar: 500 μm. (F) Percentage of tumor-free MADM-mutant mice (*n*=15) after 12 months as the endpoint, showing a median latency of 11 months. Quantiles at the bottom show a narrow spreading of tumor latency.

To overcome these limitations, we used a mouse genetic system known as mosaic analysis with double markers (MADM) developed by our laboratory (Fig. 1A). MADM consists of a pair of chimeric GFP- and RFP-coding sequences (separated by a loxP-containing intron) knocked into homologous chromosomes. Each knock-in cassette is syntenic with either the wild-type or mutant allele of one or more tumor suppressor genes on the same chromosome. From a non-labeled heterozygous animal, Cre/loxP-mediated inter-chromosomal mitotic recombination followed by X segregation of chromosomes generates a homozygous mutant cell labeled with GFP and its sibling wild-type cell labeled with RFP. Mutant cells are rare (0.1-1% or even lower) owing to the low frequency of inter-chromosomal recombination (Muzumdar et al., 2007; Zong et al., 2005), thereby approximating sporadic cancer initiation. The permanent GFP labeling of rare mutant cells enables spatially resolved investigation of their aberrant behavior at any time point during tumorigenesis (Fig. 1A) (Liu et al., 2011; Yao et al., 2020; Zong et al., 2005). Furthermore, along with a GFP^+^ mutant cell, MADM simultaneously generates a sibling RFP^+^ wild-type cell, which serves as an internal reference that enables the detection of subtle abnormalities of mutant cells. Here, we applied MADM to establish a mouse genetic mosaic model for BLBC, in which cancer initiates from sparse *Brca1, p53*-deficient cells in mammary glands. Our analyses of GFP^+^ mutant cells up to early malignancy provide important insights into cancer initiation and pre-malignant progression, and could enable future studies on the early detection and prevention of cancer.

## RESULTS

### MADM model reveals the process of mammary tumorigenesis initiated by sporadic loss of *Brca1* and *p53*

To establish a MADM-based mouse model of breast cancer, we prepared two stock mouse lines through a multi-generational breeding scheme (Fig. S1). For one stock line, we bred the *Brca1* and *p53* mutant alleles (Jacks et al., 1994; Xu et al., 1999) onto the MADM-TG allele (Hippenmeyer et al., 2010). For the other stock line, we introduced the *MMTV-Cre* transgene (Wagner et al., 2001) into the MADM-GT line (Hippenmeyer et al., 2010) to target mammary epithelial cells. Finally, we inter-crossed the two stock lines to generate MADM*-Brca1*-*p53; MMTV-Cre* mice (Fig. S1B; hereafter referred to as MADM-mutant mice), in which sparse GFP^+^ *Brca1, p53-*null cells are predisposed to becoming cancerous (Fig. 1A).

The rarity of GFP^+^ mutants in MADM not only closely mimics human cancer initiation but also enables clonal analysis of pre-malignant expansion (Greaves and Maley, 2012; Knudson, 1971; Muzumdar et al., 2007). We assessed the abundance of GFP^+^ mutant cells in mammary glands from MADM-mutant mice at 3 months of age, an age shortly after the peak in *MMTV-Cre* expression (Buono et al., 2006; Wagner et al., 1997). Using whole-mount fluorescence imaging of mammary glands, we found a high abundance of heterozygous cells (GFP and RFP double positive, which thus, appear yellow) generated from MADM recombination events in G1 or post-mitotic cells (G0) (Fig. S2A). These heterozygous cells are clearly present in the entire mammary ductal system and confirm the *in vivo* recombination by *MMTV-Cre*. Among many yellow heterozygous cells, we observed sparse and scattered GFP^+^ mutant cells along mammary ducts (Fig. 1B). For a higher-resolution view, we sectioned mammary glands and performed confocal imaging to visualize the abundance of GFP^+^ mutant cells. We found that GFP^+^ mutant cells were often singular (Fig. 1C) and accounted for ∼2% of all mammary epithelial cells, while RFP^+^ wild-type sibling cells accounted for ∼0.6% (Fig. 1D). This difference in percentage could be caused by a growth advantage of mutant cells, or a survival disadvantage of wild-type cells, or a mix of both. Notably, almost all MADM-labeled cells were positive for cytokeratin 8 (CK8; also known as KRT8), (a marker of mammary luminal cells) but negative for CK14 (a marker of mammary basal cells) (Fig. S2B-F), suggesting that they arose from the luminal layer in which the reported cell of origin for BLBC resides (Lim et al., 2009; Molyneux et al., 2010; Shehata et al., 2012). We are aware that the conventional Cre reporter showed that MMTV-Cre is expressed in both luminal and basal cells (Wagner et al., 2001). One possible explanation for this discrepancy is that MADM-labeling relies on inter-chromosomal recombination, which requires a much higher concentration of Cre than intra-chromosomal recombination for conventional floxed alleles (Zong et al., 2005). Therefore, although both luminal and basal cells express MMTV-Cre, the level could be much lower in basal cells than in luminal cells (Rabieifar, 2019), leading to luminal-specific recombination in MADM.

To assess the progression of initiated mutant cells, we collected mammary glands from MADM-mutant mice at different time points (ten mice at each age) and evaluated the overall expansion of GFP^+^ cells through whole-mount imaging. At 3 months, the expansion of GFP^+^ mutant cells was barely noticeable, but, from 6 months onward, GFP^+^ foci became visible, gradually expanded and eventually formed GFP^+^ tumors (Fig. 1E). By examining a cohort of 15 mice with an endpoint of 12 months of age, we found that 13 (87%) developed GFP^+^ tumors with a median latency of 11 months (Fig. 1F). Despite the rarity of cancer-initiating mutant cells, the MADM-mutant mice showed a tumor latency only slightly longer than the ∼9 months latency of a conditional knockout mouse model that initiates cancer with numerous mutant cells (Xu et al., 1999). This suggests that the abundance of cancer-initiating cells is not a rate-limiting factor for the kinetics of breast cancers driven by *Brca1* and *p53* deficiency. Among 43 GFP^+^ tumors from 33 mice, most mice had one to two tumors but rarely three or more tumors (Fig. S2G). Finally, we did not observe obvious tropism within the five pairs of mammary glands, except for a slightly higher incidence in the largest fourth pair and a relatively lower incidence in the second pair (Fig. S2H).

### MADM-mutant mammary tumors resemble human BLBC

**Fig. 2.**
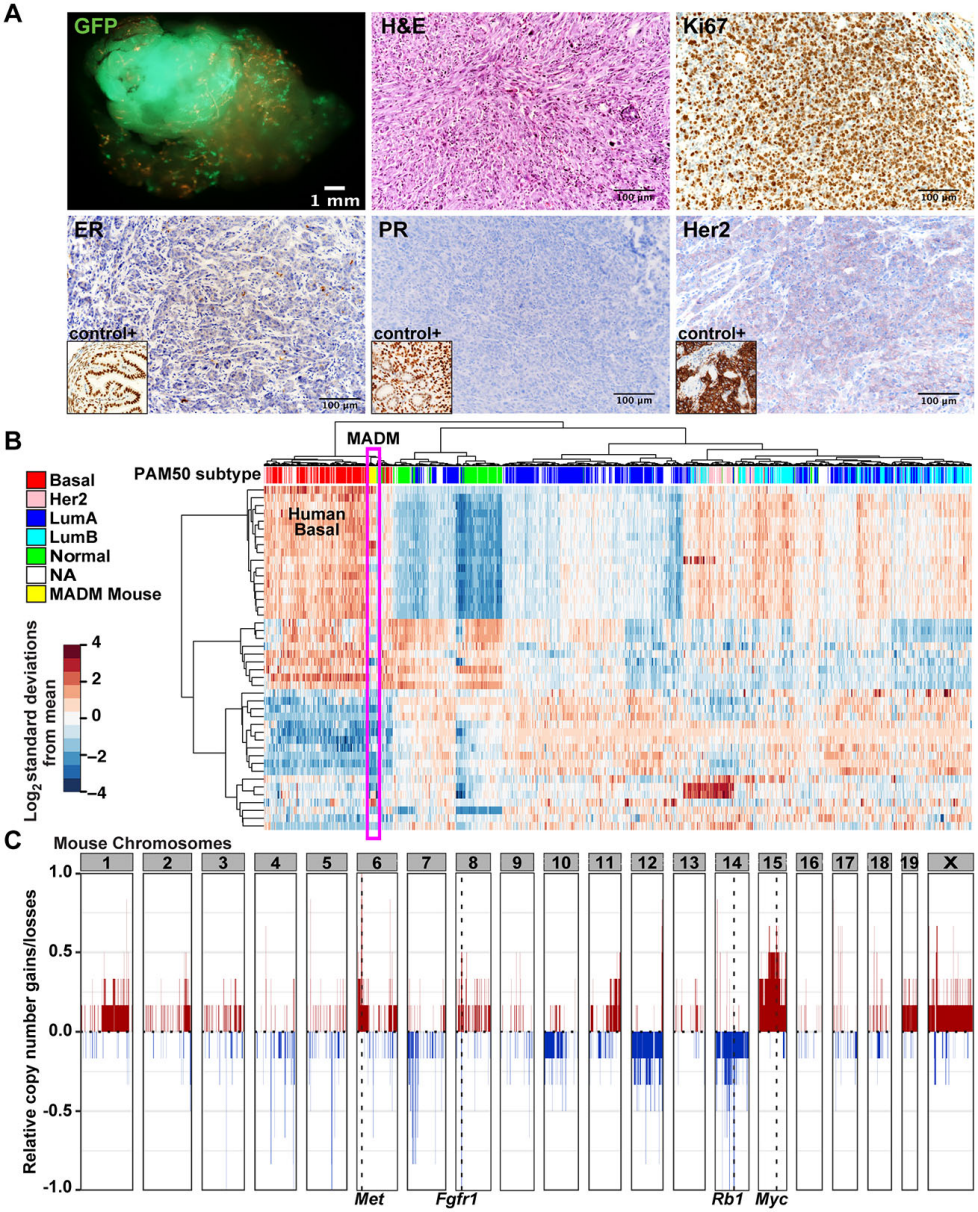
**MADM-mutant mammary tumors resemble human basal-like breast cancer.** (A) Mammary tumor with whole-mount fluorescence imaging of GFP (top left); Hematoxylin and Eosin staining (H&E; top middle); and immunohistochemistry for Ki67 (top right), estrogen receptor (ER; bottom left with a mouse oviduct as a positive control in the inset), progesterone receptor (PR; bottom middle with a mouse uterus as a positive control in the inset) and Her2 (bottom right with a mouse HER2-amplified mammary tumor as a positive control in the inset). Representative images of tumors from six mice are shown. Scale bars: 1 mm (top left) or 100 μm (other images). (B) PAM50 [a panel of 50 genes to stratify breast tumor subtypes based on Perou (2011)]-based clustering of MADM tumors with human breast cancers previously subtyped by PAM50 analysis. Twelve MADM tumors and 1104 human breast tumors from The Cancer Genome Atlas datasets were analyzed. Cross-species differences were normalized using a set of mouse-to-human orthologs (see Materials and Methods for details). (C) Copy number variations (CNVs) in six MADM tumors were assessed by whole-exome sequencing. Gains in *Met*, *Fgfr1* and *Myc*, and loss of *Rb1* are highlighted.

Human BLBCs are characterized by a high proliferation index, and lack of estrogen receptor (ER; also known as ESR1), progesterone receptor (PR; also known as PGR) and HER2 (also known as ERBB2) overexpression (Palacios et al., 2005; Perou et al., 2000; Rakha et al., 2008). We assessed six MADM tumors for proliferation and hormone receptor expression by immunohistochemistry and found them highly positive for Ki67 (also known as MKI67; ∼70% of cells) and mostly negative for ER, PR and HER2 (Fig. 2A; Fig. S3A), matching the histopathological features of human BLBCs. To further examine whether the MADM tumors resemble human BLBCs at the molecular level, we performed RNA sequencing of 12 MADM tumors and extracted a panel of 50 genes (PAM50) used to stratify breast tumor subtypes (Perou, 2011). We co-clustered PAM50 signatures of our MADM tumors with the profiles of 1104 human breast tumors from The Cancer Genome Atlas (TCGA) dataset annotated for five breast cancer subtypes. To mitigate overall differences in gene abundance across species, we identified a unique set of mouse-to-human orthologs across all TCGA and MADM tumors and normalized each sample to obtain relative expression values for each gene (see Materials and Methods for details). We found that MADM tumors clustered with the human basal-like subtype but not others (Fig. 2B). Another hallmark of human *BRCA1-*mutated BLBC is the high frequency of copy number variations (CNVs) for genomic loci containing oncogenes or tumor suppressors (Annunziato et al., 2019; Weigman et al., 2012). To determine whether MADM tumors also share this hallmark, we conducted whole-exome sequencing on six MADM tumors and paired normal somatic tissues. We found recurrent amplification of multiple chromosomal segments harboring oncogenes – such as *Met*, *Myc* and *Fgfr1* – along with recurrent deletion of the tumor suppressor gene *Rb1* (Fig. 2C), closely corresponding to human BLBCs (Fig. S3B) (Network, 2012). Collectively, the histopathological, transcriptomic and genomic analyses of MADM tumors demonstrate that our MADM-mutant mice represent an authentic model for human BLBC.

### MADM resolves the characteristic morphological stages of pre-malignant progression

**Fig. 3.**
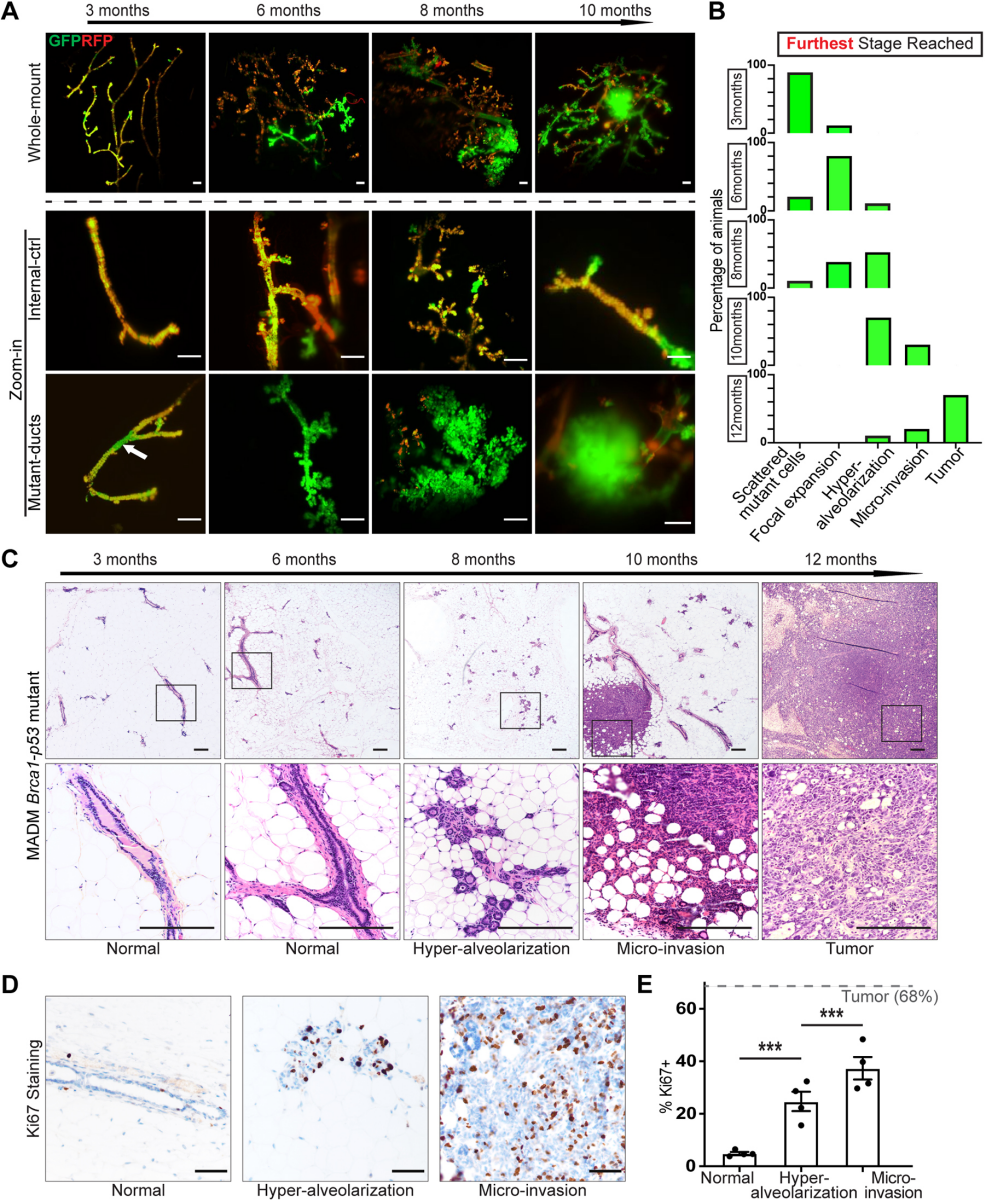
**MADM-mutant mammary glands reveal stereotyped morphological changes of pre-malignant ductal structures.** (A) Progressive morphological changes in mutant ducts (green) compared to internal control heterozygous ducts (yellow) as mice age. Top row: low-magnification 3D fluorescence imaging of mammary glands from a cohort of mice at different ages. Middle and bottom rows: higher magnifications of the control ducts and mutant ducts. Arrow indicates a GFP^+^ mutant region within the mammary ducts. Mammary glands from MADM-mutant mice at each age (*n*=10) were cleared with the CUBIC method, and 3D high-resolution images were acquired by light-sheet microscopy. Scale bars: 100 μm. (B) Timeline of the first occurrence of each pre-malignant stage of GFP^+^ mutant foci. The morphology of all mutant ducts in MADM-mutant mice at each age (*n*=10) was examined by whole-mammary-gland fluorescence imaging. Mice are categorized based on the furthest stage of pre-malignancy reached. The proportional distribution of mice at each age is plotted. (C) H&E staining of the progressive change of mammary ductal shape from normal to hyper-alveolarized, to micro-invasive, and to tumors. Paraffin slides of mammary glands from MADM-mutant mice at each age (*n*=10) were used for staining. Boxed areas in the top row are shown at higher magnification in the bottom row. Scale bars: 200 μm. (D) Ki67 staining of normal mammary ducts and MADM-mutant lesions at different stages. Ducts of each morphology were collected from four mice. Scale bars: 100 μm. (E) Quantification of percentage Ki67 positivity (% Ki67^+^) of cells within normal ducts and MADM-mutant lesions at the hyper-alveolarized and micro-invasive stages. The dashed line indicates the average % Ki67^+^ in frank tumors. Data are represented as mean±s.e.m. from *n*=4 mice. ***P<0.001 by Mann–Whitney test.

We next investigated the progressive phenotypic alterations of MADM mutant cells throughout pre-malignancy with a cohort of mice at the intermediate ages between cancer initiation and tumor formation. We performed tissue clearing with the clear, unobstructed brain/body imaging cocktails and computational analysis (CUBIC) method (Susaki et al., 2015) and then conducted whole-mount, 3D imaging using light-sheet microscopy (Fig. S4A) to look for morphological abnormalities by comparing mutant ducts with heterozygous ducts (Fig. 3A). At 3 months of age, we observed short stretches of mutant cells that occupied a continuous region without causing noticeable alterations in ductal morphology. At 6 months of age, some GFP^+^ mutant cells extended side branches resulting in a slightly more complex morphology than that of heterozygous ducts. This hyper-proliferation of mutant cells preceding prominent changes in tissue organization is consistent with observations in *BRCA1*-mutant carriers (Martins et al., 2012; McKian et al., 2009). Upon further expansion at 8 months, some mutant branches developed extensive epithelial buds reminiscent of alveologenesis during early pregnancy (Richert et al., 2000) even though these are virgin MADM-mutant mice. The alveologenesis was pervasive in late-stage mammary glands and exhibited prominently distinct histology when compared with the internal control heterozygous ducts (Fig. S4B,C). We further quantified the number of buds (alveoli) per 100-µm primary ducts and found that mutant ducts had over tenfold more alveoli than the controls (Fig. S4D,E). Binning the data into four levels based on the number of alveoli per 100 µm primary ducts – level 0 (<5), level I (5-20), level II (20-50) and level III (>50) (Fig. S4F) – we found that high-level alveologenesis (II-III) exclusively occurred within the GFP^+^ mutant regions (Fig. S4G), suggesting that this ‘hyper-alveolarization’ is a characteristic feature of pre-malignant lesions. In 10-month-old mice, we occasionally observed tiny GFP^+^ spherical masses (<1 mm in diameter) that had lost ductal morphology but were not yet palpable, which were termed as ‘micro-invasion’ hereafter (Fig. 3A).

To further determine whether the hyper-alveolarized ducts and the micro-invasions reflect a sequential progression of mammary cancer in MADM-mutant mice, we evaluated whether there is a temporal sequence in the occurrence of these structures. Because multiple expansion levels of mutant cells often co-exist in the same gland (Fig. S4B), we plotted the most-advanced GFP^+^ lesion observed in the mammary gland of each MADM-mutant animal at a series of ages, and observed a progressive emergence of focally expanded GFP^+^ cells, hyper-alveolarized GFP^+^ ducts and micro-invasions, culminating in the formation of GFP^+^ tumors at ∼1 year of age (Fig. 3B). Histological analysis further supported a temporal sequence of these pre-malignant structures (Fig. 3C). Although mammary epithelial cells from control wild-type mice appeared normal across all ages (Fig. S5A), mutant cells in hyper-alveolarized ducts present at 8 months displayed abnormal nucleomegaly (∼1.5× larger than wild-type nuclei), small but conspicuous nucleoli and loss of the basally oriented nuclear polarity (Fig. S5B). The micro-invasions contained residual hyper-alveolarized lobular units along with more advanced microinvasive carcinoma (measuring less than 1 mm) composed of individual cells and larger cords that elicited a stromal response (Fig. S5C). Finally, the frank tumors were dominated by infiltrating cells with a desmoplastic stromal response; no remnants of alveoli were visible (Fig. 3C). The progressive nature from hyper-alveolarizations to micro-invasions to full-blown tumors was further supported by Ki67 staining, revealing a gradual increase in cell proliferation (Fig. 3D,E). Thus, the MADM-mutant model revealed that cancer-initiating cells progress through a visually identifiable sequence of ductal morphology alterations before the emergence of mammary tumors.

### Hyper-alveolarized structures arise from mutant ductal rather than alveolar regions

**Fig. 4.**
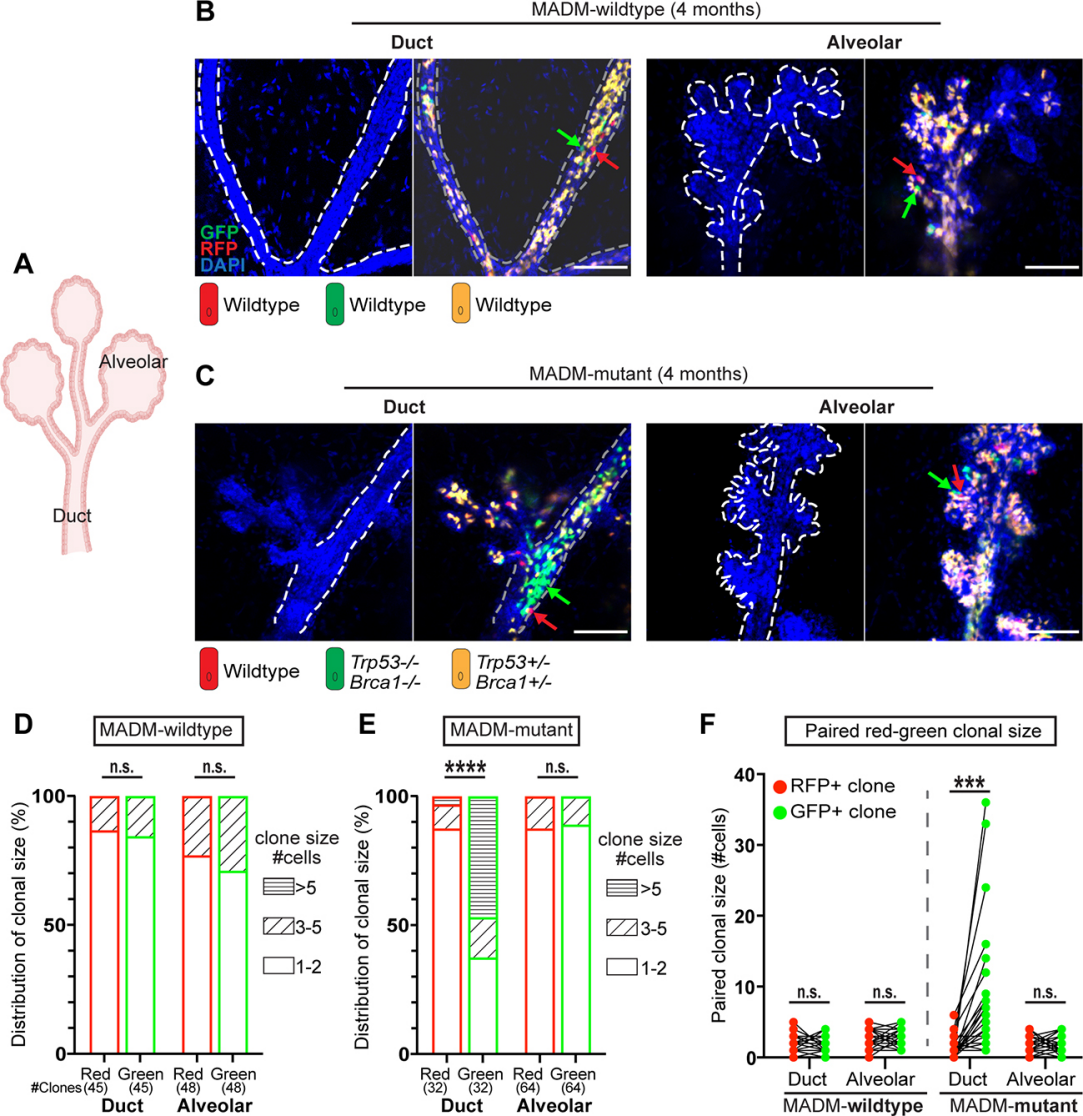
**Ductal, but not alveolar, cells show initial clonal expansion in MADM-mutant glands.** (A) Schematic of ductal and alveolar regions in the mouse mammary gland. (B) GFP^+^ and RFP^+^ clone pairs of MADM-wild-type mice are of similar sizes and do not show prominent expansion. 3D fluorescence images of cleared mammary glands from MADM-wild-type mice at 4 months of age (*n*=4) were acquired as *z*-stacks on a confocal microscope. Green arrows indicate GFP^+^ cells; red arrows indicate RFP^+^ cells. The dashed lines demarcate ductal or alveolar regions. Scale bars: 100 μm. (C) GFP^+^ mutant clones in the ductal region of MADM-mutant mice show prominent expansion, whereas their sibling RFP^+^ clones do not. In the alveolar region, neither GFP^+^ nor RFP^+^ clones expand. 3D fluorescence images of cleared mammary glands from MADM-mutant mice at 4 months of age (*n*=4) were acquired as *z*-stacks on a confocal microscope. Green arrows indicate GFP^+^ cells; red arrows indicate RFP^+^ cells. The dashed lines demarcate ductal or alveolar regions. Scale bars: 100 μm. (D) The clonal size distribution of GFP^+^ and RFP^+^ clones in ductal and alveolar regions of mammary glands from MADM-wild-type mice at 4 months of age. Data were pooled from four mice, and the total number of clones is indicated for each group. Not significant (n.s.; *P*>0.05) by Fisher's exact test. (E) The clonal size distribution of GFP^+^ and RFP^+^ clones in ductal and alveolar regions of mammary glands from MADM-mutant mice at 4 months of age. Data were pooled from four mice, and the total number of clones is indicated for each group. n.s. (*P*>0.05), ****P<0.0001 by Fisher's exact test. (F) Quantification of the clonal size for paired GFP^+^ and RFP^+^ sibling cells in the same clones in the ductal and alveolar regions of mammary glands from both MADM-wild-type and MADM-mutant mice at 4 months of age. Clones from four mice were pooled. Data are represented as mean±s.e.m. ***P<0.001 by paired two-tailed Student's *t*-test.

The mammary epithelium is composed of two anatomically and functionally distinct compartments – the alveolar regions that produce milk during lactation and the ductal regions that drain milk to the nipple (Fig. 4A) (Visvader, 2009; Visvader and Stingl, 2014). It was unclear whether the hyper-alveolarized structures consisting of mutant cells originated from alveolar or ductal mutants. Because MADM generates one RFP^+^ wild-type sibling cell alongside each original GFP^+^ mutant cell (Fig. 1A), we investigated this question using the ‘twin-spot’ analysis that directly compares mutant cells with their wild-type sibling cells in a clone-by-clone fashion within ductal and alveolar compartments, respectively (Espinosa and Luo, 2008; Muzumdar et al., 2007; Terry et al., 2020).

For the twin-spot analysis, we selected mice at 4 months of age because the focal expansion of GFP^+^ mutant cells was evident at this age, while hyper-alveologenesis was not yet present (Fig. 3B). As a baseline, we first analyzed 40 twin spots in four MADM-wild-type mice that lacked *Brca1* and *p53* mutant alleles (Fig. S1B), and confirmed that GFP^+^/RFP^+^ cells did not expand prominently and remained similar in number in both ductal and alveolar regions (Fig. 4B,D; data for each individual mouse plotted in Fig. S6A). In contrast, when we performed the twin-spot analysis in four MADM-mutant mice, we readily observed prominent expansion of GFP^+^ mutant clones over RFP^+^ wild-type clones in the ductal region (Fig. 4C,E; data for each individual mouse plotted in Fig. S6B). Surprisingly, the mutant clones in alveoli, even though harboring the same *Brca1* and *p53* mutations as those in ducts, did not expand and showed no difference in clonal size from the neighboring RFP^+^ wild-type clones (Fig. 4C,E). To conduct the twin-spot analysis more rigorously, we compared the sizes of the GFP^+^ and RFP^+^ sibling clones in a strictly pairwise manner, and found that only GFP^+^ mutant clones within the ductal regions exhibited significantly larger sizes than their RFP^+^ sibling clones (Fig. 4F), indicating that the initial clonal expansion of *Brca1, p53*-null cells occurs exclusively in the ductal region. This finding is particularly interesting because, although multiple studies with human tissues and conventional mouse models have noted the outgrowth of alveolar-like buds during the pre-malignant development of breast cancer and interpreted it as aberrant alveolar cell expansion (Bach et al., 2021; McKian et al., 2009; Poole et al., 2006; Tao et al., 2017), our results showed that such outgrowth most likely originates from luminal progenitors in the ducts that are either intrinsically fated for the alveolar lineage (i.e. alveolar luminal progenitors within the ducts) or acquire the aberrant alveolar fate due to p53/BRCA1 loss.

### The onset of luminal-to-basal transition coincides with the appearance of micro-invasion

**Fig. 5.**
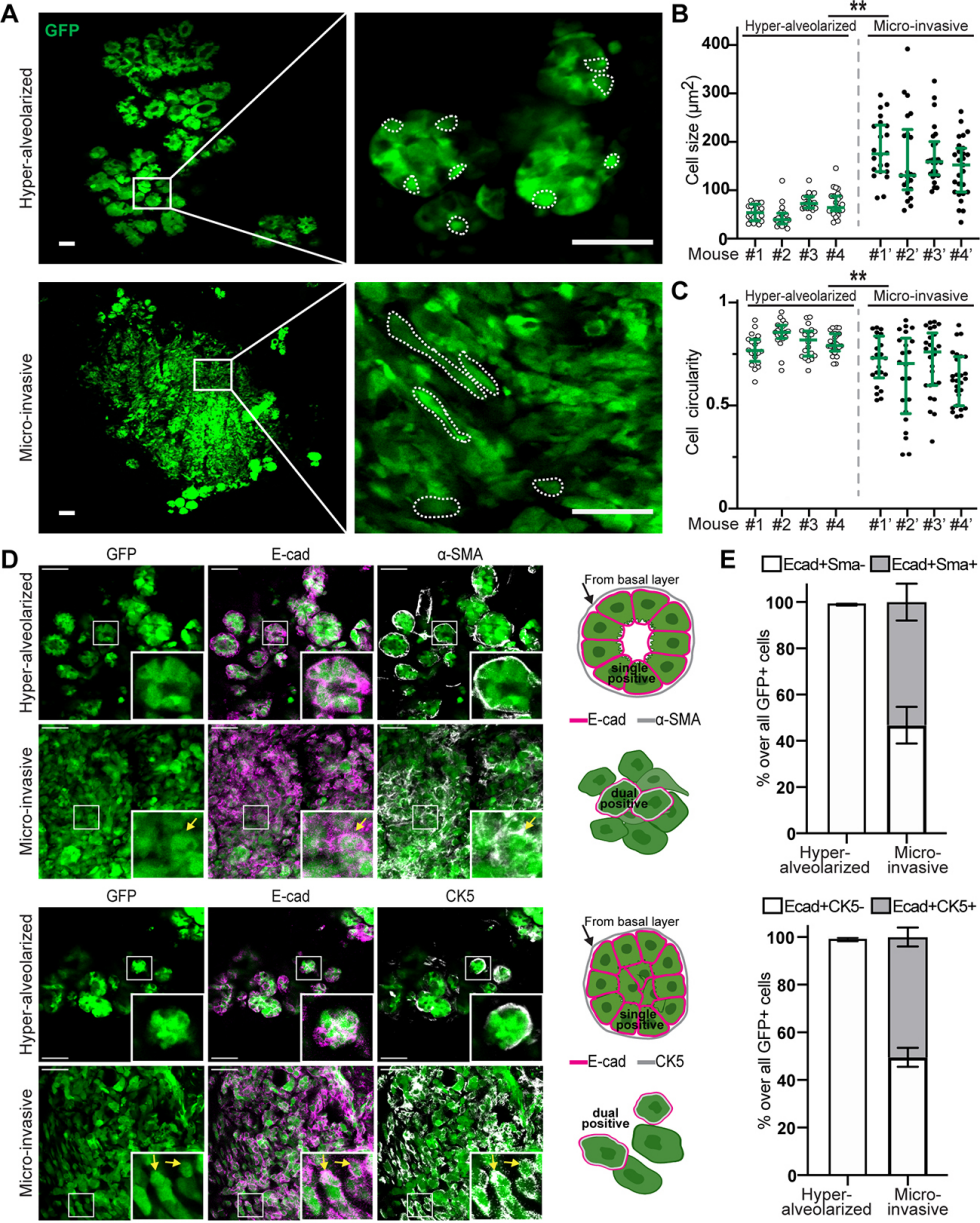
**MADM-mutant cells undergo a partial luminal-to-basal transition upon micro-invasion.** (A) Morphology of GFP^+^ mutant cells in hyper-alveolarized ducts and micro-invasive lesions. Mammary gland sections were imaged by confocal microscopy. For each feature, samples from four mice were assessed. The dashed lines delineate single cells. Scale bars: 50 μm. (B) Size of mutant cells in hyper-alveolarized ducts and micro-invasions. Cells from four mice that represent each stage were plotted for each mouse. Data are represented as median±interquartile range. **P<0.01, by nested *t*-test. (C) The circularity of mutant cells in hyper-alveolarized ducts and micro-invasions. Cells from four mice that represent each stage were plotted for each mouse. Quartiles are shown. Data are represented as median±interquartile range from *n*=50 cells. **P<0.01, by nested *t*-test. (D) In hyper-alveolarized ducts, GFP^+^ mutant cells were exclusively positive for E-cadherin (E-cad) but negative (outside of the GFP^+^ signal) for α-SMA (top panel) or CK5 (bottom panel), whereas in micro-invasions, some mutant cells were E-cad^+^α-SMA^+^ dual positive (top panel) or E-cad^+^CK5^+^ dual positive (bottom panel). Frozen sections of mammary glands were stained and imaged by confocal microscopy. For each feature, samples from four mice were analyzed. Boxed areas are shown at higher magnification in insets. Scale bars: 50 μm. (E) Top: the proportion of E-cad^+^SMA^−^ (luminal) and E-cad^+^SMA^+^ (partially transitioned) mutant cells in hyper-alveolarized ducts and micro-invasions. Bottom: the proportion of E-cad^+^CK5^−^ (luminal) and E-cad^+^CK5^+^ (partially transitioned) mutant cells in hyper-alveolarized ducts and micro-invasions. For each data point, a total of ∼700 mutant cells from mammary glands from four mice were assessed. Data are represented as mean±s.d.

BLBCs express basal-cell markers [CK5/14, α-SMA (also known as ACTA2), P63, etc.] yet arise from luminal progenitor cells (Lim et al., 2009; Liu et al., 2007; Molyneux et al., 2010; Shehata et al., 2012). This luminal-to-basal transition is thought to be critical for cancer progression, as it promotes stemness and invasiveness of *BRCA1*- or *p53*-mutant mammary epithelial cells *in vitro* (Bai et al., 2022; Kim et al., 2011; Liu et al., 2008; Mizuno et al., 2010). However, *in vivo* mapping of luminal-to-basal transitions during tumorigenesis is lacking. To determine whether luminal-to-basal transition occurs in the two representative pre-malignant stages of MADM-mutant mice (hyper-alveolarized ducts and micro-invasions), we leveraged the single-cell resolution of MADM to carefully assess the mutant cells at each stage. Normal mammary luminal cells present a cuboidal shape, whereas basal cells show an elongated spindle shape (Rios et al., 2014). Within hyper-alveolarized ducts in ∼8-month-old MADM-mutant mice, mutant cells mostly exhibited a cuboidal shape with relatively homogeneous cell size (Fig. 5A, top row). In contrast, mutant cells in micro-invasions in ∼10-month-old MADM-mutant mice showed heterogeneous morphologies, with a fraction adopting an elongated spindle shape reminiscent of basal cells (Fig. 5A, bottom row). When we quantified the cell size and circularity of mutant cells in hyper-alveolarized ducts and micro-invasions from four MADM-mutant mice, we found that the mutant cells in micro-invasions were significantly larger in size and lower in circularity than those in hyper-alveolarized ducts (Fig. 5B,C), implying a transition of cell state between these two stages.

To clarify whether the morphological change represents a luminal-to-basal transition, we assessed the expression of luminal cell marker (E-cadherin; also known as CDH1) and two basal cell markers (α-SMA, CK5) in mutant cells by immunofluorescent staining. In hyper-alveolarized ducts, GFP^+^ mutant cells maintained the expression of E-cadherin and were negative for basal markers (Fig. 5D, top and bottom panels, top rows). In micro-invasions, by contrast, some mutant cells co-expressed E-cadherin and basal cell markers (Fig. 5D, top and bottom panels, bottom rows), suggesting an incomplete transition from the luminal to basal cell state. We quantified this observation by analyzing a total of ∼700 cells in mammary tissues from four mice with hyper-alveolarized ducts or micro-invasions. In hyper-alveolarized ducts, E-cadherin-positive mutant cells were almost exclusively negative for α-SMA and CK5, but, in micro-invasions, ∼50% of mutant cells expressed both basal cell markers and E-cadherin (Fig. 5E). The incompleteness of the luminal-to-basal transition in micro-invasions was further supported by the prevalent expression of another luminal marker CK8/18 in GFP^+^ mutant cells, comparable to that within hyper-alveolarized mutant ducts (Fig. S7A). Only sporadic ER^+^ cells were found at both stages (Fig. S7B), which is consistent with the current understanding that ER^−^ luminal progenitor cells serve as the cancer cell of origin of BLBC with *BRCA1* mutations (Lim et al., 2009). Taken together, the cellular heterogeneity and the incomplete luminal-to-basal transition in micro-invasions revealed by our study likely present an excellent therapeutic opportunity and warrant further comprehensive molecular and cellular analysis.

## DISCUSSION

In this study, we established a mouse genetic mosaic model for BLBC that shares histopathological, transcriptomic and genomic similarities with human BLBCs. Taking advantage of the spatial resolution provided by MADM, we identified multiple morphologically distinct pre-malignant stages, including focal expansion of mutant cells, hyper-alveolarization of mutant ducts and micro-invasion. Surprisingly, the clonal analysis revealed that hyper-alveolarized mutant structures originate from ductal rather than alveolar cells. Further progression from hyper-alveolarized structures to micro-invasions resulted in loss of ductal organization and an incomplete luminal-to-basal transition, manifested by enlarged cell size, elongated cell shape, elevated cell proliferation and gain of basal marker expression without losing luminal marker expression. Overall, our MADM-based mouse model provides a useful tool for studying the pre-malignancy BLBC, which should empower pre-clinical research on early detection and cancer prevention.

MADM-based cancer models offer several unique advantages. First, MADM creates a small number of homozygous mutant cells within a heterozygous animal, closing mimicking the sporadic loss of heterozygosity (Lohmussaar et al., 2020) of tumor suppressor genes in cancer. For germline *BRCA1* mutation carriers, cancer is often initiated by the sporadic loss of the wild-type *BRCA1* allele (Cornelis et al., 1995; Maxwell et al., 2017; Nones et al., 2019). Second, the generation of mutant cells by MADM is coupled with permanent GFP labeling through a mitotic recombination event, allowing mutant cells to be unequivocally identified and tracked throughout the entire process of tumorigenesis (Muzumdar et al., 2007; Zong et al., 2005). Because both GFP and tdTomato are expressed at the high enough level to be visible under a fluorescence stereomicroscope within fresh, unstained mammary glands (Fig. 1E), MADM enables the gross evaluation of pre-malignant phenotypes and targeted sample collection prior to various downstream analyses that require unfixed tissues. Third, MADM generates sibling wild-type cells that are labeled with RFP, providing an internal control for the GFP^+^ mutant cells (Beattie et al., 2017; Terry et al., 2020). Without RFP^+^ wild-type siblings as a reference (Fig. 4), it would be difficult to distinguish the clonal expansion of GFP^+^ mutant ductal cells from stochastic neutral drift (Lopez-Garcia et al., 2010; Snippert et al., 2010). Although our study is focused on breast cancer modeling, it should be noted that, owing to the modular nature of the MADM system and the availability of a genome-wide library of MADM mice (Contreras et al., 2021), one could establish many other cancer models to study their initiation and pre-malignant progression upon the sporadic LOH of relevant tumor suppressor genes.

The observed progression trajectory in the MADM-mutant mice provided multiple insights for understanding the early genesis of BLBC. Although hyper-alveolarization was initially thought to reflect the aberrant expansion of mutant alveolar cells (Tao et al., 2017), our study showed that it actually originates from mutant ductal cells either intrinsically fated for or misdifferentiated toward the alveolar fate upon *p53* and *BRCA1* mutations. Corroborating our finding, the recent single-cell sequencing of mammary glands from *Brca1, p53*-deficient mice spanning various pre-malignant ages revealed dysregulation of transcription factors driving alveologenesis in luminal progenitor cells, causing aberrant alveolar outgrowth (Bach et al., 2021). Intriguingly, this finding may point to distinct roles of hormonal signaling between pre-malignancy and malignancy of BLBC: the hyper-alveolarization of mutant ducts resembles alveolar outgrowth during early pregnancy (Macias and Hinck, 2012), a process known to be regulated by the progesterone signaling (Brisken et al., 1998; Humphreys et al., 1997; Lydon et al., 1995); and progesterone receptors are overexpressed in *Brca1*-deficient mammary epithelial cells of human and mouse, and exposure to exogenous progesterone dramatically increases mammary gland volume in *Brca1*-deficient mice (King et al., 2004; Ma et al., 2006; Poole et al., 2006). Therefore, although the expression of hormonal receptors is low/absent in malignant BLBCs, progesterone signaling could play significant roles at pre-malignancy, which warrants further study and may offer a new avenue of cancer prevention (Nolan et al., 2016; Sigl et al., 2016; Trabert et al., 2020).

At the stage of micro-invasion, MADM-mutant cells undergo a partial luminal-to-basal transition, which could increase the stemness and invasiveness of *Brca1*- or *p53*-deficient cells, as reported in the literature (Bai et al., 2022; Kim et al., 2011; Liu et al., 2008; Luond et al., 2021; Mizuno et al., 2010), and was recently implicated as a critical step at the onset of basal-like tumorigenesis (Landragin et al., 2022 preprint). Our time-course analysis of *Brca1, p53*-deficient cells *in vivo* showed that the loss of *Brca1* and *p53* does not immediately induce a luminal-to-basal transition even after the manifestation of hyper-alveolarization. Instead, mutant cells only reach an incomplete luminal-to-basal transition at ∼10 months after mutant cells progress to micro-invasive lesions. Although we cannot rule out cell-intrinsic mechanisms for this transition, our observation implies that the exposure of luminal cells to extrinsic stromal factors due to basement membrane breaching in micro-invasion could be the trigger for the luminal-to-basal transition, which should be investigated more thoroughly in the future.

Although powerful, MADM has certain limitations. First, the tumor latency tends to be long, e.g. it took ∼8 months for MADM-mutant mice to progress into the pre-malignant stages in this study. If desired, additional clinically relevant mutations could be introduced to accelerate cancer development in our model (Annunziato et al., 2019). Compound mutations that are syntenic with *Brca1* and *p53* can be introduced using the same scheme as shown in Fig. S1B; for mutations that are not syntenic, mutant alleles can be introduced into the MADM model through conventional breeding schemes (Muzumdar et al., 2016; Yao et al., 2020). Second, owing to the involvement of many genetic elements in the MADM model, it tends to have mixed genetic background that precludes allotransplantation experiments. When necessary, one could backcross stock mice into desired pure genetic background to improve the versatility of this model. Third, MADM relies on mitotic recombination to generate mutant cells, and thus cannot be used to mutate post-mitotic cells. Finally, when constitutively expressed Cre transgene is used, e.g. MMTV-Cre for this study, the birth timing of mutant cells is not clear. When desired, one could use temporally controlled Cre lines, such as Tet or CreER system, to precisely control the timing of tumor initiation. Notwithstanding these limitations, the current MADM model for BLBC enables spatially resolved analysis throughout the pre-malignant progression, and can greatly facilitate studies of early detection and cancer prevention.

## MATERIALS AND METHODS

### Animal

The following mouse lines were crossed to establish the MADM-mutant and MADM-wild-type mice: TG11ML (stock #022976, The Jackson Laboratory) (Henner et al., 2013), GT11ML (stock #022977, The Jackson Laboratory) (Henner et al., 2013), *Brca1^flox^* (strain #01XC8, NCI Mouse Repository) (Xu et al., 1999), *p53^KO^* (stock #002101, The Jackson Laboratory) (Jacks et al., 1994), *MMTV-Cre* (stock #003553, The Jackson Laboratory) (Wagner et al., 1997). The breeding schemes are shown in Fig. S1. We exclusively used female mice, primarily focusing on the fourth pair of mammary glands (the abdominal pair) for data collection, unless otherwise specified in figure legends. All animal work was performed in the University of Virginia Animal Vivarium. All procedures, including housing and husbandry were approved by the Institutional Animal Care and Use Committee (IACUC) at the University of Virginia, following national guidelines to ensure the humanity of all animal experiments.

### Genotyping

For genotyping, the mouse toe was used to extract DNA for PCR. First, 120 µl of 50 mM NaOH was added to each toe and then incubated at 95°C for 20 min in the PCR machine, followed by addition of 30 µl of 1 M Tris-HCl (pH 7.4) and mixing. One microliter of the toe solution was used for the PCR template in a 20 µl PCR reaction. PCR primer sequences were as follows. (1) MADM TG/GT cassettes: primer-1, 5-TGGAGGAGGACAAACTGGTCAC-3; primer-2, 5-TCAATGGGCGGGGGTCGTT-3; primer-3, 5-TTCCCTTTCTGCTTCATCTTGC-3; PCR products, knock-in (KI) band, 230 bp and wild-type band, 350 bp. (2) *MMTV-Cre*: primer-1, 5-CACCCTGTTACGTATAGCCG-3; primer-2, 5-GAGTCATCCTTAGCGCCGTA-3; PCR product, KI band, 300 bp. (3) *p53^KO^*: primer-1, 5-ACCGCTATCAGGACATAGCGTT-GG-3; primer-2, 5-CACAGCGTGGTGGTACCTTATG-3; primer-3, 5-GGTATACTCAG-AGCCGGCCTG-3; PCR products, KI band, 700 bp and wild-type band, 450 bp. (4) *Brca1^flox^*: primer-1, 5-CTGGGTAGTTTGTAAGCATCC-3, primer-2, 5-TCTTATGCCCTCAGAAAACTC-3; PCR products, flox/flox band, 365 bp and wild-type band, 297 bp.

### Immunofluorescence and immunohistochemistry

Mammary glands were harvested and fixed with 4% paraformaldehyde (PFA) at 4°C for 24 h. For immunofluorescence, tissues were then washed with PBS twice, soaked with 30% sucrose at 4°C for 48 h, and embedded in optimal cutting temperature (OCT) compound (SAKURA). The tissues were sectioned at 20 µm thickness with a Thermo Fisher Scientific NX50 Cryostat. For staining, slides were first blocked in 0.3% Triton-X 100 and 5% normal donkey serum in PBS for 20 min, then incubated with primary antibodies (anti-CK8, Abcam, ab182875, 1:200; anti-CK14, BioLegend, 905301,1:400; anti-E-cadherin, BioLegend, 147301, 1:200; anti-α-SMA, Sigma-Aldrich, A5228, 1:500) diluted in blocking buffer at 4°C overnight. Secondary antibody incubation was performed for 1 h at room temperature in PBS. To stain nuclei, slides were incubated in 4′,6-diamidino-2-phenylindole (DAPI) solution (1 µg/ml in PBS) for 5 min before being mounted with 70% glycerol. Fluorescent images were acquired on Zeiss LSM 700/710 confocal microscope. Images were processed with Zen and Fiji. For immunohistochemistry, PFA-fixed tissue was further processed for paraffin embedding and then sectioned at 4 µm thickness. After antigen retrieval, the primary antibody (Ki67, Epitomics, 4203-1, 1:400) was incubated at 4°C overnight. Horseradish peroxidase (HRP)-conjugated secondary antibodies were then used, and 3,3′-diaminobenzidine (Vector Laboratories, SK-4100) was used to develop color.

### Tissue clearing with the CUBIC method and 3D imaging

PFA-fixed mammary glands were cleared for large-scale 3D imaging with the standard CUBIC method (Susaki et al., 2015). Briefly, tissues were immersed in 50% reagent-1 (25 wt% urea, 25 wt% Quadrol, 15 wt% Triton X-100, 35 wt% dH_2_O), shaken at 110 rpm at 37°C for 12 h, and then transferred to 100% reagent-1 with DAPI (1 µg/ml) for shaking until mammary glands became transparent. After reagent-1, tissues were washed three times with PBS, 1 h each time, with shaking to remove reagent-1. Tissues were then shaken in 50% reagent-2 for 12 h at 37°C, followed by 100% reagent-2 (25 wt% urea, 50 wt% sucrose, 10 wt% triethanolamine, 15 wt% dH_2_O), shaking for 48 h. A Zeiss Z.1 light-sheet microscopy system was used for acquiring images. Tissues were attached to the holder of the light-sheet microscope with super glue.

### Gene expression profiling

Approximately 50 mg tissue from each mammary tumor was used for RNA extraction. The tissue was homogenized in 500 µl TRlzol, then 100 μl chloroform was added and mixed thoroughly, followed by centrifugation (12,000 rcf) for 15 mins at 4°C. The upper-layer aqueous phase containing the RNA was transferred to a new tube, and 5 μg polyacrylamide was added, followed by an equal volume of 70% ethanol. Afterward, 700 μl of the sample was used as the input for RNA isolation using an RNeasy Mini Kit (Qiagen) according to the manufacturer's protocol. The quality of RNA samples was evaluated with Bioanalyzer (Agilent Technologies), and samples with an RNA integrity number (RIN) >8 were used for library preparation. Libraries were prepared with a TruSeq Stranded mRNA Library Prep kit (Illumina). Libraries were multiplexed at an equimolar ratio, and 1.3 pM of the multiplexed pool was sequenced on a NextSeq 500 instrument with a NextSeq 500/550 Mid/High Output v2.5 kit (Illumina) to obtain 75-bp paired-end reads. From the sequencing reads, adapters were trimmed using fastq-mcf in the EAutils package (version ea-utils.1.1.2-779) with the following options: −q 10 −t 0.01 −k 0 (quality threshold 10, 0.01% occurrence frequency, no nucleotide skew causing cycle removal). Quality checks were performed using FastQC (version 0.11.8) and MultiQC (version 1.7). Data were aligned to the murine transcriptome (GRCm38.84) using HISATv2 (version 2.1.0) with options for paired-end reads. HISAT read counts were converted to transcripts and normalized to transcripts per million (TPM) using StringTie (version 2.1.6).

### PAM50 extraction and comparison with TCGA datasets

TCGA breast cancer expression data were obtained from the UCSC genome browser (Ciriello et al., 2015). Human orthologs for mouse genes were obtained from the Ensembl biomart in R using the getAttributes function. For the human and murine datasets, we obtained the intersection of unique orthologs to obtain a set of 14,980 mice to human ortholog genes to evaluate co-expression. To enable cross-species comparisons, we performed a sample-wise column normalization to obtain new TPM estimates that accounted for gross differences in gene abundance between species. Hierarchical clustering of PAM50 genes was performed using ‘pheatmap’ in R using Euclidean distance and ‘ward. D2’ linkage.

### Whole-exome sequencing and CNV analysis

Genomic DNA was prepared from tumors developed in MADM*-*mutant mice and from the tails of the same mice as the control with a DNeasy Blood and Tissue Kit (Qiagen). Whole-exome sequencing at 100× coverage was performed as a contract service with Genewiz. Raw BCL files were converted to fastq files with bcl2fastq v.2.19, and adapter was trimmed with Trimmomatic v.0.38. Trimmed reads were mapped to the mouse reference genome, and somatic variants and CNVs were called using the Dragen Bio-IT Platform (Illumina) in somatic mode and a panel of normals to remove technical artifacts. The filtered VCF was annotated with Ensembl Variant Effect Predictor (VEP) v95 for the Ensembl transcripts overlapping with the filtered variants. CNVs that passed quality control filters from Dragen were visualized using the GenVisR v.1.16.1 package in R.

### Statistical analysis

Statistical analysis was performed with GraphPad Prism. Bar graphs were presented as the mean±s.e.m. unless otherwise annotated in figure legends. The normality of data distribution was checked with qqplot in R. Depending on whether the data followed a normal distribution, paired two-tailed Student's *t*-test or Mann–Whitney *U* test was used as indicated in the figure legends. The Chi-square test or Fisher's exact test was used to test frequency distribution as indicated in the figure legends. Statistical significance is denoted by ‘not significant (n.s.)’ (*P*>0.05), **P<0.01, ***P<0.001 and ****P<0.0001.

## Supplementary Material

10.1242/dmm.050219_sup1 Supplementary information
