# Supplementary material for: A genetic mosaic mouse model illuminates the pre-malignant progression of basal-like breast cancer
Source: Dis Model Mech. 2023 Nov 13;16(11):dmm050219. doi: 10.1242/dmm.050219 (PMC10668031; doi:10.1242/dmm.050219)
Supplement: Supplementary information [file dmm-16-050219-s1.pdf]

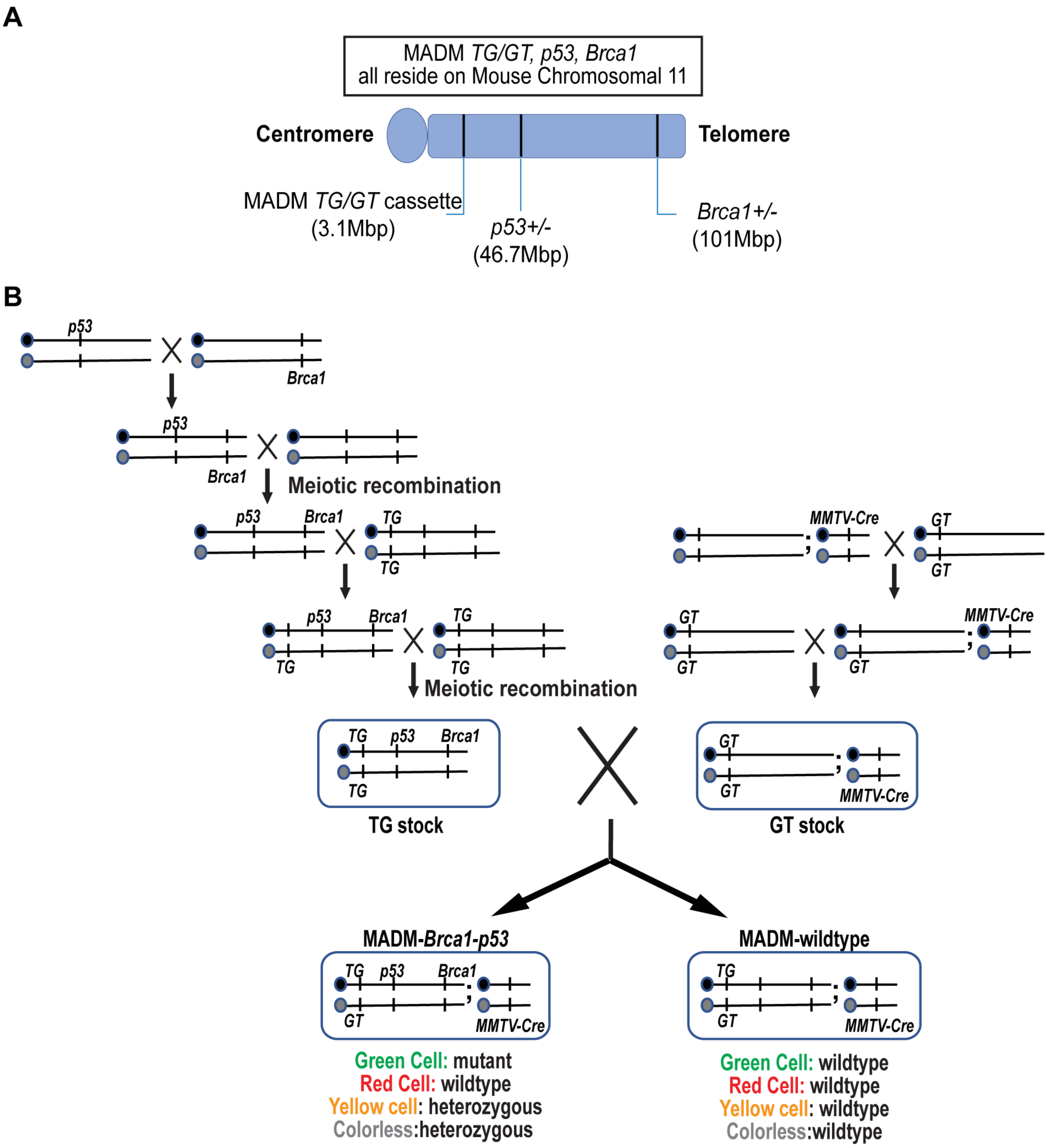

Disease Models & Mechanisms • Supplementary information

Fig. S1. The breeding scheme to build two stock mouse lines for a MADM-based breast cancer mouse model with *Brca1, p53* deficiency

(A) Location of MADM TG/GT cassettes, *p53*, and *Brca1* on mouse chromosome 11. The physical locations were indicated.

(B) The breeding scheme to incorporate *p53* and *Brca1* mutations into MADM-TG stock and the *MMTV-Cre* transgenes into the MADM-GT stock. Mating between the TG and GT stock produces MADM *p53-Brca1* mice (MADM mutant) and the control MADM wildtype mice.

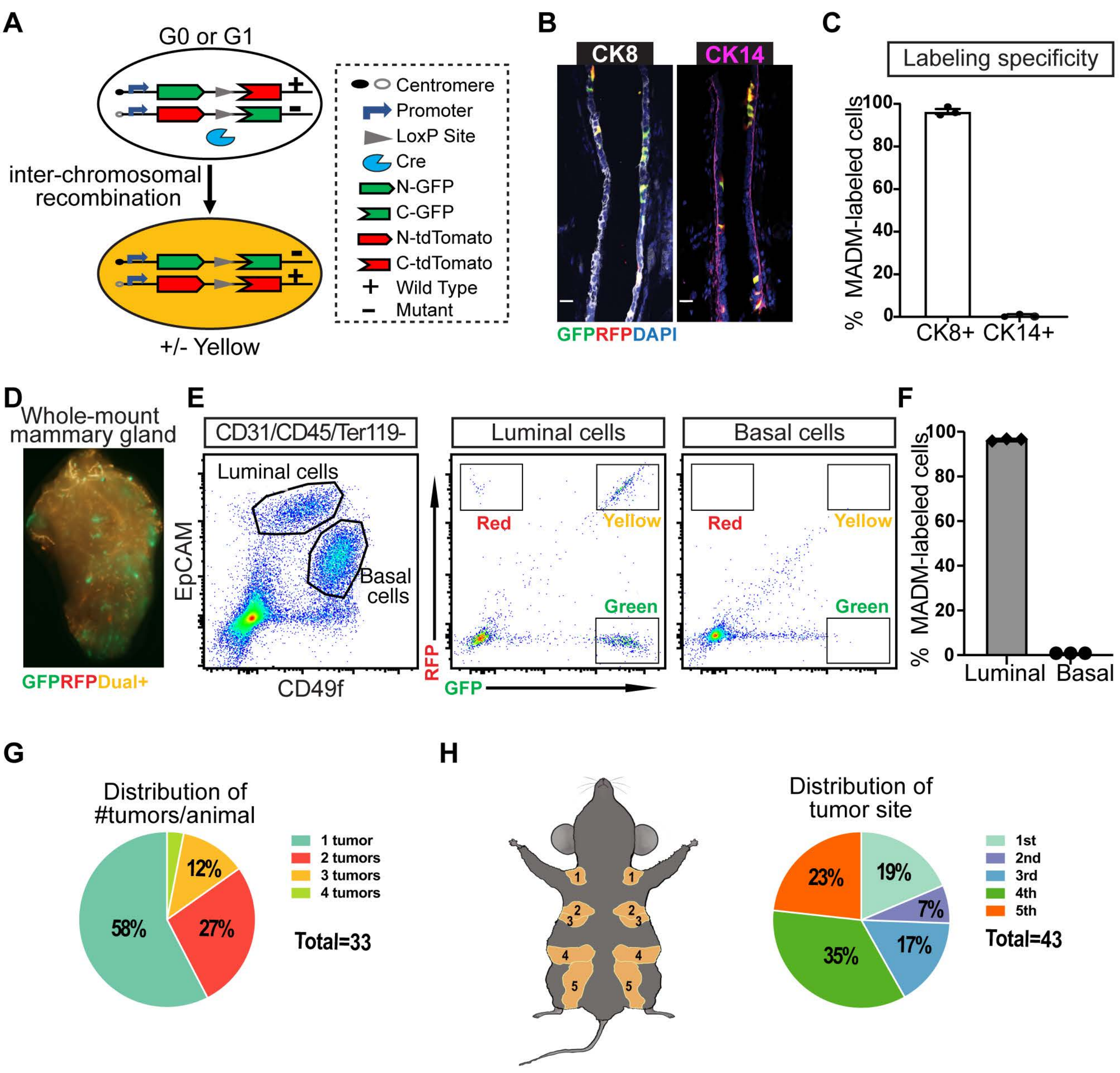

Disease Models & Mechanisms • Supplementary information

Fig. S2. Additional mechanism to generate yellow cells, MADM labeling specificity in the luminal cells, and distribution of tumor among glands.

(A) Cre-mediated inter-chromosomal recombination could also occur in G1 or post-mitotic cells (G0), which generate dual-colored yellow cells without altering genotype (heterozygous).

(B) MADM-colored cells locate in the CK8+ luminal layer but not in the CK14+ basal layer. Immunofluorescence staining of CK8 and CK14 was performed with sections of mammary glands from MADM-mutant mice at three months old (*n* =3). Scale bar =50 μm.

(C) Quantification of the percentage of MADM-colored cells that are CK8+ (luminal) or CK14+ (basal) from three MADM-mutant mice at three months old (*n* =3). Data are represented as mean ± s.d.

(D) Representative whole-mount fluorescence image of MADM mammary glands used for disassociation and flow analysis. Three mice at ~ 10 months old were used.

(E) MADM-colored cells were predominantly found in luminal cells but not in basal cells.

(F) The percentage of MADM-labeled cells that are luminal or basal cells. Each dot represents data from one mouse (n =3). Data are presented as mean ± s.d.

(G) The total number of tumors per mouse among a total of 33 MADM-mutant mice.

(H) The distribution of tumors among 5 pairs of mammary glands, based on the assessment of a total of 43 GFP+ tumors from 33 MADM-mutant mice.

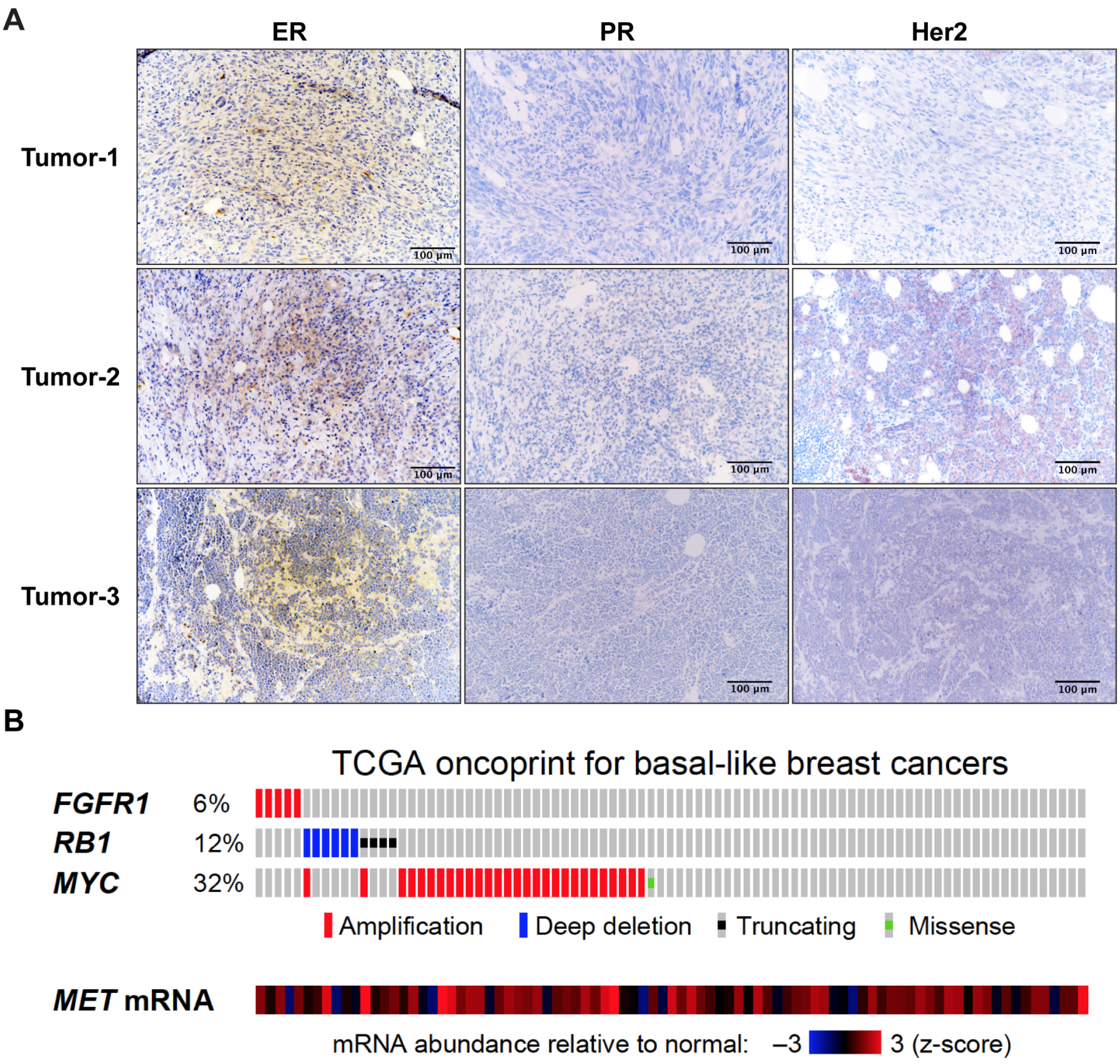

Disease Models & Mechanisms • Supplementary information

Fig. S3. The hormone receptor status of MADM tumors and CNVs in human basal-like breast cancer

(A) Representative images of ER, PR, and Her2 status in three more MADM tumors showed a triple-negative phenotype. Immunohistochemistry staining was performed with tumor sections. Scale bar =100 μm.

(B) Analysis with TCGA datasets for human basal-like breast cancer, showing consistent amplification of *FGFR1, MYC*, loss of *RB1*, and over-expression of *MET*.

Disease Models & Mechanisms • Supplementary information

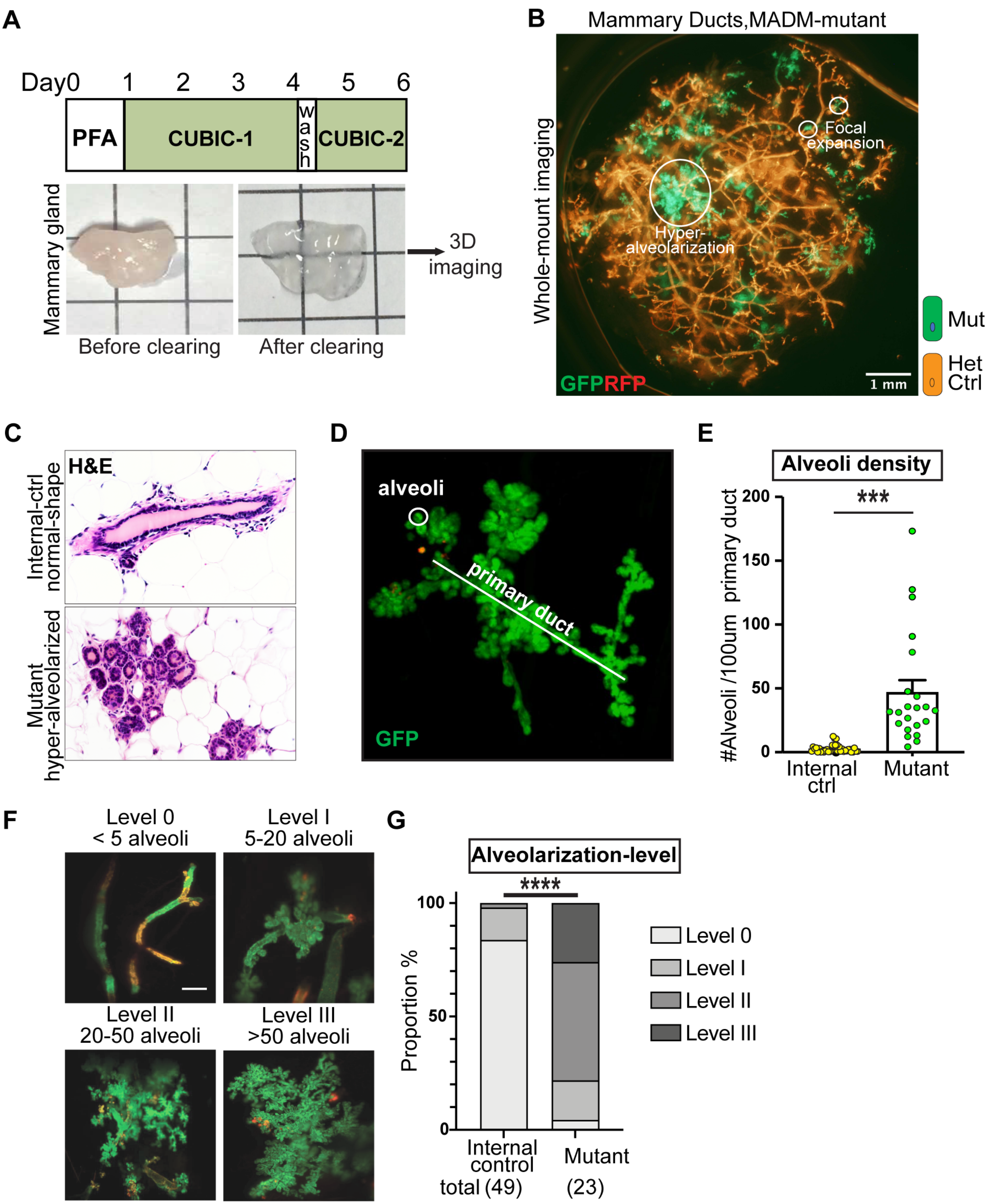
A
Day0 1 2 3 4 5 6
PFA
CUBIC-1
wash
CUBIC-2
Mammary gland
Before clearing
After clearing
3D imaging
B
Mammary Ducts,MADM-mutant
Whole-mount imaging
Focal expansion
Hyper-alveolarization
GFPRFP
1 mm
Mut
Het Ctrl
C
H&E
Internal-ctrl normal-shape
Mutant hyper-alveolarized
D
alveoli
primary duct
GFP
E
Alveoli density
***
#Alveoli /100um primary duct
200
150
100
50
0
Internal ctrl
Mutant
F
Level 0
< 5 alveoli
Level I
5-20 alveoli
Level II
20-50 alveoli
Level III
>50 alveoli
G
Alveolarization-level
****
Proportion %
100
50
0
Internal control
Mutant
total (49)
(23)
Level 0
Level I
Level II
Level III

Fig. S4. Hyper-alveologenesis occurred specifically in mutant ducts.

(A) The pipeline for acquiring large-scale 3D images of mammary ducts with the CUBIC clearing method and light-sheet microscopy.

(B) Whole-mount fluorescence imaging of cleared mammary glands showing distinct morphology of mutant ducts. Mammary glands from 4 MADM-mutant mice at 8 months old mice were assessed. Scale bar =100 μm.

(C) H&E staining of hyper-alveolarized mutant ducts and normal-shape control ducts.

(D) The scheme to count the number of alveoli per 100 μm major ducts to quantify ductal-alveologenesis.

(E) Increased ductal-alveologenesis level of the green mutant ducts compared with the yellow heterozygous ducts (internal control) as shown by the quantification of ductal alveologenesis level from 8-month-old mice (n=4). Data are represented as mean ± s.e.m. Mann–Whitney test was used, ***<0.0001.

(F) Examples and criteria for categorizing mutant ducts into different alveologenesis levels.

(G) The distribution of ductal-alveologenesis levels among yellow heterozygous ducts (internal control) and green mutant ducts in glands from 8-month-old mice (n=6). 49 yellow control ducts and 23 green mutant ducts were imaged and quantified. A Chi-square test was used. **** p<0.0001.

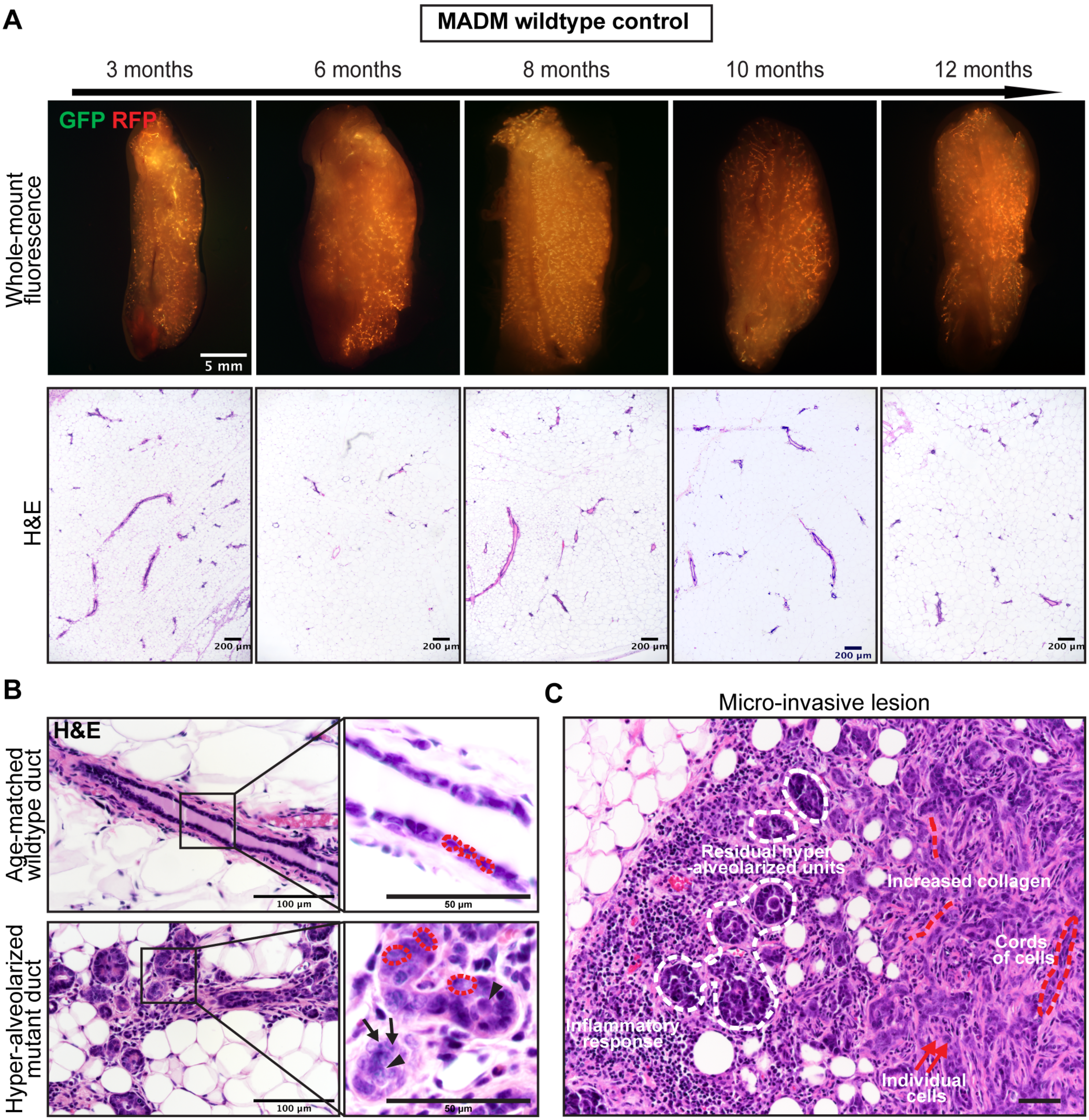

Disease Models & Mechanisms • Supplementary information

Fig. S5. Morphology of mammary ducts in MADM-wildtype mice along aging, and detailed pathological features of hyper-alveolarized mutant ducts and micro-invasive lesions.

(A) Upper panel: whole-mount fluorescence imaging of mammary glands from MADM-wildtype mice at a cohort of ages, showing no expansions of GFP+ foci or prominent morphological change of mammary ducts. Scale bar =5 mm. Lower panel: H&E staining showing no morphological changes of mammary ducts along mice aging. Scale bar =200 μm. *n*=3 for each age.

(B) H&E staining of hyper-alveolarized mutant ducts and age-matched wildtype ducts. Mutant cells in hyper-alveolarized ducts exhibited 1) abnormal nucleomegaly, with nuclei approximately 1.5 times larger than those of wildtype cells (dashed circles), 2) small yet conspicuous nucleoli (arrows), indicative of proliferation, and 3) loss of the basolateral axis of nuclear polarity in some cells (arrowheads). Representative images from four mice. Scale bar =100 μm.

(C) H&E staining of micro-invasive lesions. The micro-invasive lesions encompass residual hyper-alveolarized units (white dashed circles), alongside with more advanced micro-invasive carcinoma (on the right), measuring less than 1 mm. Within these lesions, individual cells (red arrows) and cords of cells lacking the lumen (red dashed circles) invade the adipose area and elicit a stromal response marked by increased collagen deposition (red dashed lines, intense eosin stains). Inflammatory responses are evident at the border. Representative images from four mice. Scale bar =100 μm

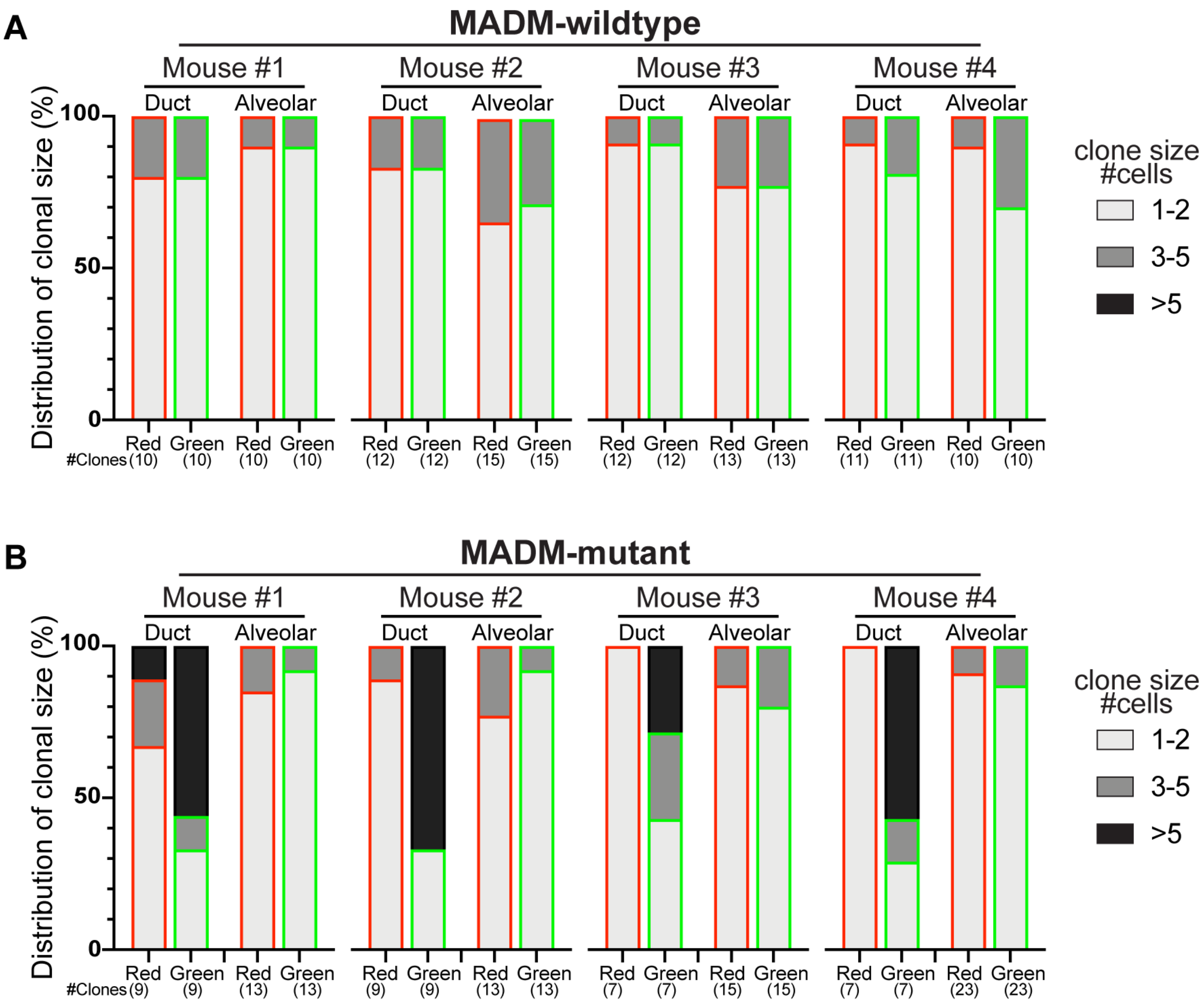

Disease Models & Mechanisms • Supplementary information

Fig. S6. Size distribution of GFP+ and RFP+ clones in ductal and alveolar regions of mammary glands from individual MADM-wildtype and MADM-mutant mice.

(A) Size distribution of GFP+ and RFP+ clones in ductal and alveolar regions of mammary glands in four individual MADM-wildtype mice.

(B) Size distribution of GFP+ and RFP+ clones in ductal and alveolar regions of mammary glands in four individual MADM-mutant mice.

*The total number of clones is indicated in parenthesis at the bottom.

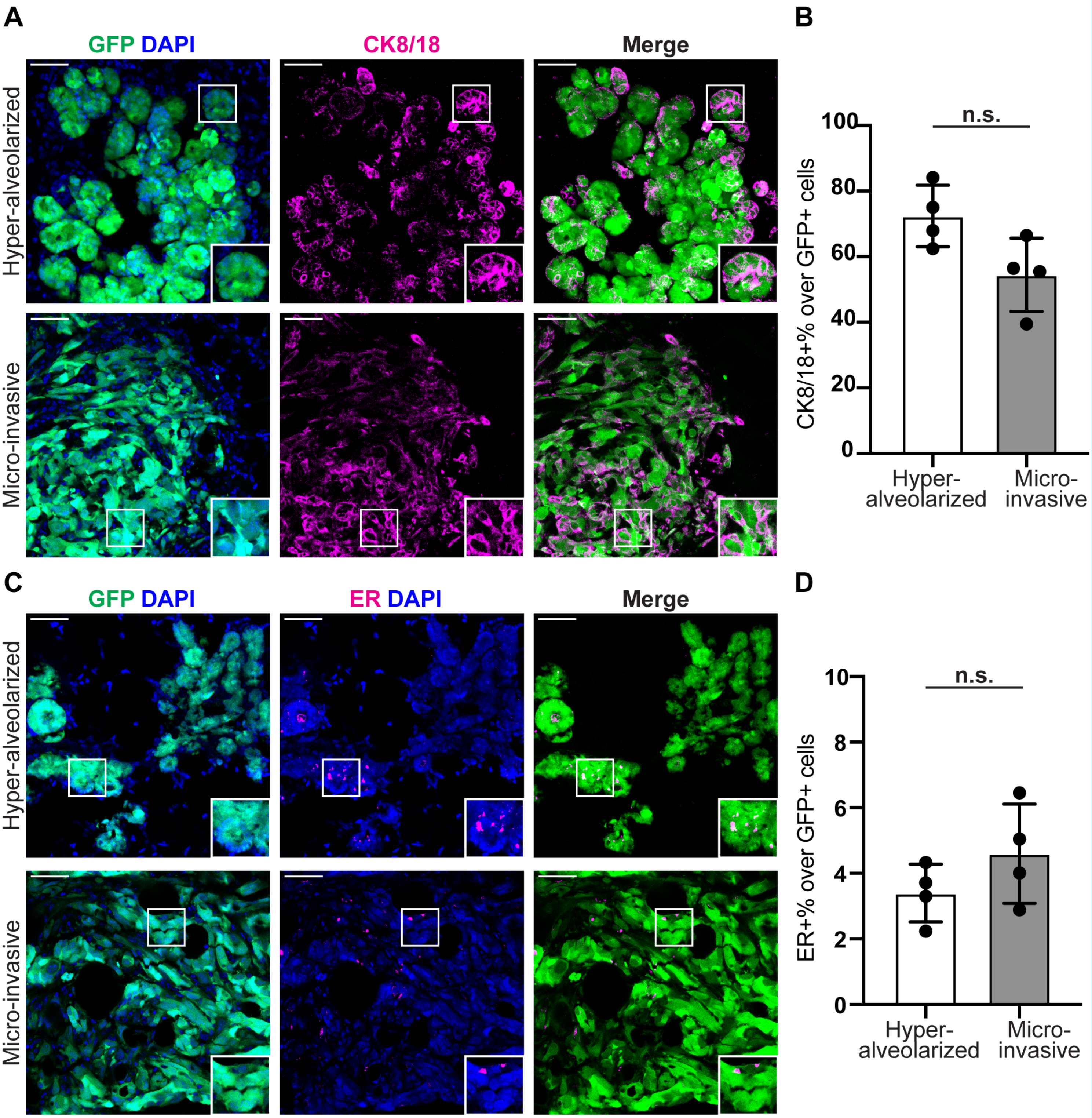

Disease Models & Mechanisms • Supplementary information

Fig. S7. CK8/18 and ER staining of hyper-alveolarized mutant ducts and micro-invasive lesions.

(A) Cells in both hyper-alveolarized ducts and micro-invasive lesions are mostly CK8/18+. Frozen sections of mammary glands from four mice that represent each stage were stained and imaged by confocal microscopy. Scale bar =50 μm.

(A) The proportion of CK8/18+ cells among all GFP+ mutant cells. Each dot represent data from one mice. For each mouse, ~1000 cells from four imaging fields were quantified. Data are represented as mean ± s.d., n.s. >0.05 by Mann–Whitney test.

(B) Only sporadic ER+ cells are present in hyper-alveolarized ducts and micro-invasive lesions. Frozen sections of mammary glands from four mice that represent each stage were stained and imaged by confocal microscopy. Scale bar =50 μm.

(C) The proportion of ER+ cells among all GFP+ mutant cells. Each dot represent data from one mice. Data are represented as mean ± s.d., n.s. >0.05 by Mann–Whitney test.

Disease Models & Mechanisms • Supplementary information
